# Fortifying a meal with oyster mushroom powder beneficially affects postprandial glucagon-like peptide-1, non-esterified free fatty acids and hunger sensation in adults with impaired glucose tolerance: a double-blind randomized controlled crossover trial

**DOI:** 10.1007/s00394-021-02674-1

**Published:** 2021-09-10

**Authors:** Lisa Dicks, Linda Jakobs, Miriam Sari, Reinhard Hambitzer, Norbert Ludwig, Marie-Christine Simon, Peter Stehle, Birgit Stoffel-Wagner, Hans-Peter Helfrich, Jenny Ahlborn, Martin Rühl, Bolette Hartmann, Jens J. Holst, Sabine Ellinger

**Affiliations:** 1 Department of Nutrition and Food Sciences, Niederrhein University of Applied Sciences, Mönchengladbach, Germany; 2 Department of Nutrition and Food Sciences, Human Nutrition, University of Bonn, Meckenheimer Allee 166a, 53115 Bonn, Germany; 3 Department of Nutrition and Food Sciences, Nutrition and Microbiota, University of Bonn, Bonn, Germany; 4 Department of Nutrition and Food Sciences, Nutritional Physiology, University of Bonn, Bonn, Germany; 5 Institute of Clinical Chemistry and Clinical Pharmacology, University Hospital Bonn, Bonn, Germany; 6 Institute of Numerical Simulation, University of Bonn, Bonn, Germany; 7 Institute of Food Chemistry and Food Biotechnology, University of Giessen, Giessen, Germany; 8 NNF Center for Basic Metabolic Research and Department of Biomedical Sciences, University of Copenhagen, Copenhagen, Denmark

**Keywords:** Oyster mushrooms, *β*-Glucans, Postprandial glucose and lipid metabolism, Gastrointestinal hormones, Appetite sensations, Impaired glucose tolerance

## Abstract

**Purpose:**

Impaired glucose tolerance (IGT) is a pathophysiological condition characterized by insulin resistance with known metabolic consequences such as postprandial hyperglycemia and hypertriglyceridemia. We hypothesized that fortifying a meal with mushrooms rich in *β*-glucans may diminish glucose and triglyceride responses by improving postprandial gastrointestinal hormone release.

**Methods:**

In a randomized controlled crossover study, 22 subjects with IGT ingested a meal either enriched with 20 g powder (8.1 g *β*-glucans) of oven-dried *Pleurotus ostreatus* (enriched meal, EN) or without enrichment (control meal, CON). Blood was collected before and repeatedly within 4 h after the meal to determine AUC of glucose (primary outcome), insulin, triglycerides, non-esterified free fatty acids (NEFAs), glucagon-like peptide-1 (GLP-1), gastric inhibitory polypeptide (GIP) and ghrelin. Appetite sensations (hunger, satiety, fullness, and desire to eat) were assessed before and after meal consumption by visual analog scales.

**Results:**

Postprandial glucose, insulin, triglycerides, GIP and ghrelin concentrations as well as the corresponding AUCs did not differ between EN and CON. NEFAs-AUC was 14% lower (*P* = 0.026) and GLP-1-AUC 17% higher (*P* = 0.001) after EN compared to CON. Appetite ratings did not differ between treatments, except for hunger (AUC 22% lower after EN vs. CON; *P* = 0.031).

**Conclusion:**

The observed immediate postprandial metabolic changes indicate that an easily manageable fortification of a single meal with powder from dried oyster mushrooms as *β*-glucan source may improve postprandial metabolism. If the effect is preserved long term, this measure can diminish the risk for further development of overweight/obesity and type 2 diabetes in subjects with IGT.

**Clinical trial registration:**

German Clinical Trial Register on 09/08/2018; trial-ID: DRKS00015244.

**Supplementary Information:**

The online version contains supplementary material available at 10.1007/s00394-021-02674-1.

## Introduction

Impaired glucose tolerance (IGT) is a pathophysiological condition characterized by insulin resistance leading to postprandial hyperglycemia and hypertriglyceridemia [1]. These metabolic consequences are often accompanied by an altered secretion of gastrointestinal (GI) hormones, especially of incretins such as glucagon-like peptide-1 (GLP-1) occurring in reduced plasma concentrations [2]. GLP-1 increases insulin secretion, delays gastric emptying (GE) and acts as signal to induce satiety [3]. Adults with IGT are often overweight or obese and show, compared to glucose tolerant adults, a 5% higher risk of progression to diabetes mellitus per year [4] as well as an approximately 30% higher risk to develop composite cardiovascular diseases (CVD), thereby increasing the 9 year all-cause mortality [5]. A key component in the treatment of IGT is practicing a balanced, energy-controlled diet to normalize postprandial blood glucose (PBG) levels and to regulate body weight (BW) [6]. An essential part of dietary measures to reach these goals is an adequate intake of dietary fibers; cereal *β*-glucans [linear polysaccharides built from D-glucopyranosyl units linked via *β*-(1,3) and *β*-(1,4) bonds [7]] have especially been shown to be effective [8]. In the GI tract, cereal *β*-glucans generate highly viscous solutions, thereby delaying GE, slowing down the digestion of dietary carbohydrates (CHO), like starch, and retarding subsequent absorption of monosaccharides released [8–10]. The latter, in turn, modulates the secretion of GI hormones, such as ghrelin, GLP-1 and gastric inhibitory polypeptide (GIP) [11]. Consequently, the European Food Safety Authority (EFSA) has published health claims qualifying *β*-glucans from oats or barley consumed together with available carbohydrates (avCHO; 4 g *β*-glucans/30 g avCHO) as an evidence-based measure to reduce PBG and to increase/prolong post-meal satiety [8]. Regular intake of cereal fiber [12, 13] and oats [14] significantly reduced glycosylated hemoglobin A1c, fasting blood glucose [14] and BW [13], thus leading to a 18% lower risk of CVD mortality [12].

Although commonly used in food design, fortification of dishes with *β*-glucan-rich cereal products such as oat flakes or oat bran (2.6–8.2 g *β*-glucans/100 g dry matter (DM) [15–18]) is restricted by their limited palatability and their avCHO content (42.7–48.5/100 g DM [15, 16] corresponding to 5.2–18.6 g avCHO per gram *β*-glucan). Additional consumption of avCHO is unfavorable for subjects with IGT and may be contrary to the intended fortification goals. Edible mushrooms are also rich in *β*-glucans, especially *Pleurotus* species (spp.) (24.2–35.0/100 g DM [19, 20]), but provide less avCHO (19.7–59.3 g avCHO/100 g DM [21, 22]) per gram *β*-glucan (0.5–2.4 g avCHO/g *β*-glucan). As shown in vitro [23], mushroom *β*-glucans have a similar capability to slow down starch digestibility and the subsequent release of glucose as cereal *β*-glucans despite their slightly different chemical structure (glucopyranosyl units are cross-linked via *β*-(1,6) bonds to the linear *β*-(1,3) main chain [7]). In a randomized controlled trial (RCT), the enrichment of a single meal with 5 g *Pleurotus eryngii* (*P. eryngii*) extract reduced lipemic response in hyperlipidemic, non-diabetic subjects compared to the non-enriched meal [24]. The partial replacement of wheat flour with *Pleurotus sajor-caju* (*P. sajor-caju*) powder in biscuits reduced their glycemic index up to 17% [25]. Moreover, a single supplement of dried *Pleurotus ostreatus* (*P. ostreatus,* 50 mg/kg BW) 30 min before intake of 75 g pure glucose lowered 2 h PBG in patients with type 2 diabetes mellitus (T2DM), probably due to increased insulin release [26]. However, human intervention studies evaluating the overall metabolic effects of mushroom-fortified meals are still lacking.

Therefore, the aim of this RCT in subjects with known IGT was to investigate whether fortifying a usual meal with powder from whole *P. ostreatus* fruiting bodies improves postprandial plasma appearance of glucose (primary outcome), lipemic response, the release of GI hormones and appetite sensations.

## Methods

This trial was performed between 12/2018 and 02/2019 at the Department of Nutrition and Food Sciences at Niederrhein University of Applied Sciences, Mönchengladbach, Germany.

### Participants

Participants with IGT [i.e., blood glucose ≥ 140 and < 200 mg/dL 2 h after a 75 g oral glucose tolerance test (OGTT) (ADA/WHO)] were recruited between 09/2018 and 01/2019 by in-house postings at Niederrhein University of Applied Sciences (poster, flyer, newsletter), public announcements (poster, flyer, newspaper) and postings in medical practices (poster, flyer) in the Mönchengladbach area. IGT have been medically diagnosed during the past 12 months. In other subjects interested in participation, the risk for prediabetes/T2DM was assessed by questionnaire (Diabetes Risk Test [27]) and thereafter, those identified at risk were invited for an OGTT at the research department (Accu-Chek Dextrose O.G-T., Roche, Mannheim, Germany). Criteria for eligibility were checked by questionnaire; exclusion criteria included smoking, any self-reported medication that might affect glucose metabolism (e.g., cortisone), antibiotic treatment in the past 6 weeks, diseases associated with diarrhea or malabsorption, allergies or intolerances towards ingredients of the test meal, planned changes in lifestyle, vegetarian diet, regular intake of psyllium, guar, oats, mushroom extract or apple pectin, pregnancy, lactation, present/former alcohol or drug abuse and participation in another trial. In premenopausal women, pregnancy was excluded using a urine-based pregnancy test (KTX7, Innovita Biological Technology, Tangshan, China).

### Study design and intervention

**Fig. 1:**
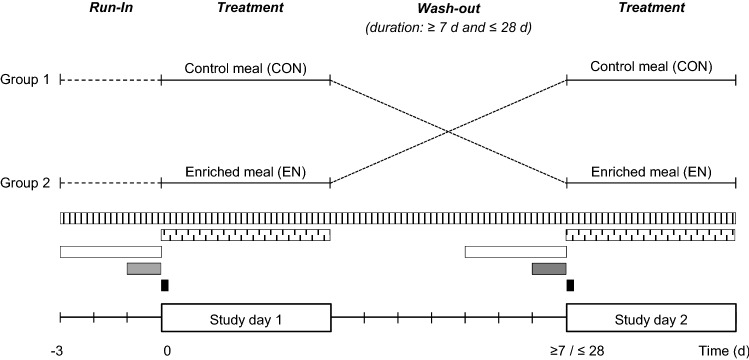
Study design. Bars reflect measures to ensure comparable conditions on both study days. Stripes: instruction not to change body weight and lifestyle; dashed: treatment of premenopausal women in the same menstrual cycle phase; white: dietary restrictions, carbohydrate intake > 150 g/day; grey: request to avoid physical exertion and to consume a comparable dinner on both pre-study days; black: arriving at study center without great physical effort and using the same means of transport

This RCT was a double-blind, acute crossover study with two treatments in random order: a CHO-based meal either enriched with powder from *P. ostreatus* (enriched meal, EN) or without enrichment (control meal, CON). These meals were provided on two different study days that were separated by at least a 1 week washout. The time course of the study is shown in Fig. 1.

After enrollment, the participants were consecutively allocated to groups 1 and 2 (determining the meal order, i.e., first CON, then EN, or vice versa) by permuted block randomization (ratio 1:1, block size of 4) using sealed envelope^™^ software (Sealed Envelope, London, UK). EN and CON were encoded as A and B; this assignment was carried out by an external person. The assignment list was stored in an opaque envelope until the statistical analysis had been concluded to ensure blinding of the researchers to treatment.

Premenopausal women were investigated in the same menstrual cycle phase and/or had to be under stable treatment with hormonal contraceptives to rule out cycle-dependent effects on target parameters [28]. Moreover, participants were instructed to maintain their BW and individual lifestyle (diet, physical activity) during the entire study period. Three days before each intervention, participants were asked to ingest ≥ 150 g CHO daily and to abstain from *β*-glucan-rich foods (e.g., mushrooms, oats) and alcoholic beverages following a detailed written guideline. Participants documented their food and beverage consumption throughout these periods using standardized 3-day food records. Subjects were instructed to avoid physical exertion (e.g., arduous gardening, endurance sports) and to have dinner before 8.00 pm on both pre-study days. Each participant was asked to eat before the second study day a dinner comparable to the one consumed before the first study day. On both study days, the participants arrived in the study center between 7.30 and 8.00 am after at least 12 h of overnight fast, using the same means of transport.

Before onset of interventions, anthropometric parameters were investigated and the compliance with pre-intervention lifestyle instructions was checked by means of a guided interview and 3 day food records; these were also checked for plausibility. For repeated blood sampling (before and 15, 30, 45, 60, 90, 120, 180 and 240 min post-consumption), an intravenous catheter was fixed. After each blood sampling, appetite sensations and adverse effects were assessed. The participants consumed the smoothie within 5 min and subsequently the soup within 10 min (see below). During the 4 h supervision period, the participants rested in a sitting position and ingested 100 mL of water every 30 min.

### Meal preparation and composition

The participants consumed a two-course meal composed of a smoothie “apple-mango-passion fruit” (250 mL; Rewe, Cologne, Germany; same lot per participant) and a creamy potato soup (390 mL; Erasco, Lübeck, Germany; lot no. 735.1907). The test meal was enriched with 20 g (equal parts added to the smoothie and the soup) oven-dried mushroom powder (*P. ostreatus;* BIO Pleurotus, Wohlrab, Entrischenbrunn, Germany; lot no. 4644) corresponding to a usual serving size of 200 g fresh *P. ostreatus* [29]. Addition of mushroom powder increased the viscosity of the smoothie and the soup at 37 °C by 142% and 53%, respectively (Advanced Rheometer AR 550, TA Instruments, New Castle, Delaware, USA). This measure did not change the volume of the smoothie and the soup as determined for a sample (1/5 of the serving size each) using a graduated cylinder (Blaubrand^©^, Duran^©^, Brand^©^, Wertheim, Germany; 100 mL; graduation 1 mL, reading accuracy 1 cm^3^ according to DIN EN ISO 4787:2011-05; data not shown). Energy and nutrient supply from each meal are presented in Table 1.

**Table 1:** Energy content and main ingredients of the meals, based on the analyses of their components^a^

	Meal components			Meal	
	Potato soup (390 mL)	Smoothie (250 mL)	Mushroom powder (20 g)	Enriched^b^	Control^c^
Energy, kcal^d^	287	116	53	456	403
Protein, g^e^	4.1	0.9	2.1	7.1	5.0
Fat, g^f^	21.3	0.2	0.3	21.8	21.5
Total carbohydrates, g^g^	23.8	28.7	15.3	67.9	52.5
Available carbohydrates, g^h^	16.0	26.5	5.5	47.6	41.1
Trehalose, g^i^	NA	NA	4.2	4.2	NA
Other α-glucans, g^j^	NA	NA	0.9	0.9	NA
Dietary fiber, g	7.9^k^	2.3^k^	9.9^l^	20.0	10.1
* β*-Glucans, g^j^	NA	NA	8.1	8.1	NA
Chitin, g^m^	NA	NA	1.7	1.7	NA
Minerals, g^n^	5.2	0.7	0.8	6.7	5.9
Moisture, g (%)^o^	335.6 (86.0)	219.5 (87.8)	1.5 (7.3)	556.5 (84.3)	555.1 (86.7)

*NA* not analyzed

^a^Data are mean values calculated from analyses in duplicate except for *β*-glucans which were investigated in quadruplicate

^b^Calculated as sum of potato soup, smoothie and mushroom powder except for trehalose, *β*-glucans, and chitin

^c^Calculated as sum of potato soup and smoothie

^d^Determined according to regulation (EU) No 1169/2011 with the exception that the protein content was calculated as described in footnote e

^e^The true protein content was calculated by the sum of analyzed amino acids, taking into account the loss of water due to the formation of peptide bonds [31]

^f^Determined according to the procedure of Weibull-Stoldt [32]

^g^Estimated by the difference method: 100 − protein − fat − minerals − moisture

^h^Calculated by subtraction of dietary fiber from total carbohydrates

^i^Determined photometrically using Trehalose Assay Kit K-TREH 12/19 (Megazyme Int. Ireland, Bray, Country Wicklow, Ireland)

^j^Determined photometrically by *β*-Glucan (Yeast & Mushroom) Assay Kit K-YBGL (Megazyme Int. Ireland) according to Sari et al. [30]

^k^Determined using total dietary fiber kit 1129790001 (Merck, Darmstadt, Germany) by a combination of enzymatic and gravimetric methods

^l^Calculated as sum of *β*-glucans and chitin

^m^Determined colorimetrically as described previously [31]

^n^Determined according to §64 of the German Food and Feed Code by ashing the sample at 550 °C

^o^Determined by thermogravimetric analysis

For blinding reasons, the smoothie was generally served in a semi-transparent covered cup with a drinking straw. The color of the non-enriched soup was adapted by adding 0.8 mL/portion of an aqueous solution of caramel color, providing 467 mg sulfite ammonia caramel E 150d (Felix Koch Couleur & Karamel, Offenbach, Germany). To imitate the fungal-like smell, 1-octen-3-ol (Silesia, Neuss, Germany) was added to the control meal (soup: 6 µL, smoothie: 10 µL). Both meals were prepared by students and were served on trays sprinkled with 1 µL of 1-octen-3-ol (Merck, Darmstadt, Germany).

### Mushroom powder

According to the manufacturer, the mushroom powder was prepared from pure, whole oven-dried fruiting bodies of *P.* *ostreatus* which had been cultivated on a substrate of cottonseed hull (88%, wt/wt), wheat bran (10%, wt/wt), sugar cane (1%, wt/wt) and CaCO_3_ (1%, wt/wt). The powder provided 8.1 g of *β*-glucans per 20 g, determined using the *β*-Glucan (Yeast & Mushroom) Assay Kit (Megazyme Int. Ireland, Bray, County Wicklow, Ireland) as described by Sari et al. [30] and 8.0 g avCHO, the major source being trehalose. Further details on the composition of the mushroom powder are provided in Table 1 and in Supplemental Table 1 [31, 32].

### Blood sampling and preparation

Venous blood was collected into tubes (Monovette^®^, Sarstedt, Nümbrecht, Germany) coated with EDTA (analysis of ghrelin, GLP-1, GIP) or NaF and citrate (plasma glucose analysis) and in tubes without anticoagulant (analysis of insulin, triglycerides (TGs), and non-esterified free fatty acids (NEFAs)). Blood samples were placed on ice immediately after sampling and centrifuged (2500 × *g*, 10 min, 4 °C) to obtain plasma; serum was collected after 20 min of clotting. All specimens were frozen at − 80 °C. After completion of the study, samples were transported on dry ice to laboratories in Bonn and Copenhagen for analysis.

### Laboratory investigations

Glucose, insulin and TGs were analyzed at the Department of Clinical Chemistry and Clinical Pharmacology, University Hospital Bonn, Germany. Glucose and TGs were investigated photometrically with cobas^®^ c 702 and insulin by ECLIA with cobas^®^ e 801 (both from Roche/Hitachi, Mannheim, Germany) using test kits (GLUC3, CV 1.1%; TRIGL, CV 0.5%; Elecsys Insulin, CV 0.9%). Glucose and insulin values were used to calculate HOMA-IR. NEFAs were investigated in duplicate at the Department of Nutrition and Food Sciences, Nutritional Physiology, University of Bonn, Germany, by means of a colorimetric test kit (HR Series NEFA-HR(2), Fujifilm Wako Chemicals Europe, Neuss, Germany) (CV 8.4%). GLP-1, GIP and ghrelin were determined at the Department of Biomedical Sciences, University of Copenhagen, Denmark. For analyses of GLP-1 and GIP, an in-house radioimmunoassay (RIA) was used. For this, all samples were extracted in a final concentration of 70% ethanol before measurement. Total GLP-1 was measured as described by Ørskov et al. [33] using a RIA (antibody code no. 89390) specific for the C-terminal domain of the GLP-1 molecule and which reacts equally with intact GLP-1 and the primary (N-terminally truncated) metabolite. Total GIP concentration was analyzed with a RIA using an antibody directed towards the C-terminal domain (code no. 80867), which reacts fully with intact GIP and N-terminally truncated forms as described previously [34]. Sensitivity for these RIAs was < 1 pmol/L, and CV < 10%. Ghrelin was determined using the Human Ghrelin (total) ELISA kit (EZGRT-89K; Millipore, Billerica, MA, USA) according to the manufacturer’s instructions. All samples from the same individual were measured in the same assay run and quality controls provided by the manufacturer were within allowed limits.

### Appetite sensations

Hunger, satiety, fullness and desire to eat were measured before and repeatedly after finishing the meal by means of separate visual analog scales according to Flint et al. [35] using an electronic digital caliper (kwb Germany, Stuhr, Germany; accuracy of 0.02 mm).

### Adverse effects

Adverse effects, such as nausea, headache and flatulence, were also assessed (questionnaire) during the supervision period.

### Anthropometric investigations

BW, height, waist and hip circumference and fat mass (FM) were investigated under standardized conditions: BW and height were used to calculate BMI; waist-to-hip ratio (WHR) was determined to classify fat distribution according to WHO. Bioelectric impedance analysis (50 kHz, 800 µA) was performed to determine FM using the equation of Kyle et al. [36].

### Dietary intake assessment

The intake of energy and selected nutrients was calculated using the software Prodi^®^ 6.7.0.0 (Nutri-Science, Freiburg, Germany).

### Sample size calculation

At the time of study planning, no trials evaluating the postprandial effect of fortifying a meal with oyster mushrooms on glucose incremental area under the curve (iAUC, primary outcome) were available. Due to the comparable effect of cereal and mushroom *β*-glucans on starch digestibility in vitro [23], we assumed similar effects on PBG. Thus, our sample size calculation was based on data obtained within a RCT with crossover design monitoring the glycemic response of overweight/obese adults to a meal (50 g avCHO) with or without a preload of oat bran (water-based drink containing 0.9, 2.6, and 5.3 g *β*-glucans and 0.6 g, 2.3 g and 4.6 g avCHO, respectively; control:water) [37]. The intake of oat bran *β*-glucans was linearly related to a decrease in glucose iAUC. Using the regression formula of Steinert et al. [37], potential effects of pre-defined *β*-glucan quantities on iAUC can be predicted: a fortification of our meal with mushroom powder providing at least 5.3 g *β*-glucans was expected to decrease iAUC by 23.1% compared to CON. To detect a mean decrease in glucose iAUC of 23.1% with a SD of 20.1% (corresponding to an effect size of 1.1), ten participants per group were needed presuming a power of 80% and an alpha of 0.05 (own calculation based on [38]). Assuming a dropout rate of 15%, 11 subjects were included in each group.

### Statistical analysis

Metric data were checked for normal distribution using the Shapiro–Wilk test and were logarithmized if necessary. If normal distribution could be assumed, parametric tests were used. Values obtained before both meals (e.g., nutrition status) were compared with each other by paired *t* test or Wilcoxon test. Repeated-measures ANOVA was performed with time and treatment as “within” factors to evaluate the effects of *P. ostreatus* enrichment on postprandial response. Otherwise, the Friedman test was used. In case of significant changes, equal time points between both treatments were compared by Wilcoxon test considering Bonferroni correction for multiple comparisons. In addition, postprandial response was quantified as total area under the curve (tAUC), except for glucose and insulin for which iAUC is recommended as this is more sensitive when distinguishing between foods with different glycemic effects [39]. If single values were missing for a participant after one treatment, the AUC was still calculated without this value. In this case, the AUC for the other treatment was calculated for this participant analogously. AUCs were compared for both treatments using paired *t* test, applying the bootstrapping option with 1000 replications if normality failed. Differences indicated by *P* values < 0.05 were considered statistically significant. For Bonferroni adjustment, original *P* values were multiplied with the number of time points (*n* = 9) to reduce the false-discovery rate. Nominal variables were compared between the treatments by Fisher’s Exact test.

Metric data are presented as means ± SEMs, unless otherwise indicated. Differences between AUCs obtained after EN and CON are provided as means and 95% CIs. Nominal and ordinal data are given as frequencies. Statistical analysis was done using IBM-SPSS Statistics 25.0 (IBM Corp., Armonk, NY, USA).

## Results

**Fig. 2:**
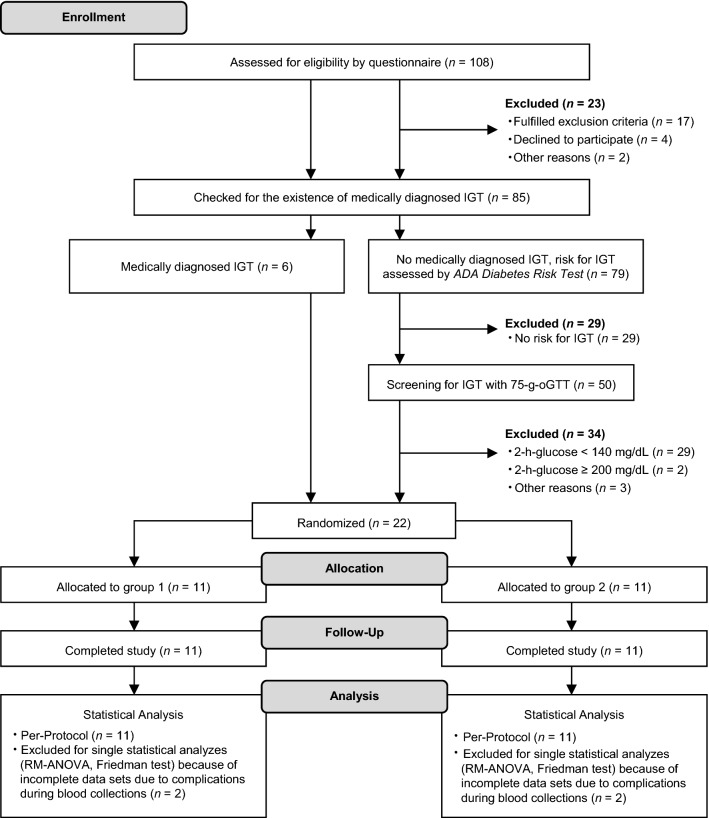
Flow of participants. ADA, American Diabetes Association; IGT, impaired glucose tolerance; OGTT, oral glucose tolerance test; RM-ANOVA, repeated-measures ANOVA

In total, 108 individuals were screened for eligibility, of whom 23 were excluded (*n* = 17 based on exclusion criteria, *n* = 4 declined to participate, and *n* = 2 due to other reasons; Fig. 2). Of the remaining 85 subjects, 6 presented a medical diagnosis of IGT and were included. Fifty subjects showed an increased risk for IGT and were asked to perform an OGTT. Of these, 16 subjects had a 2 h glucose concentration ≥ 140 and < 200 mg/dL and were, therefore, included. All 22 participants finished the study per protocol. In four subjects, blood sampling was incomplete; they were partly excluded from statistical evaluation (repeated-measures ANOVA or Friedman test).

**Fig. 3:**
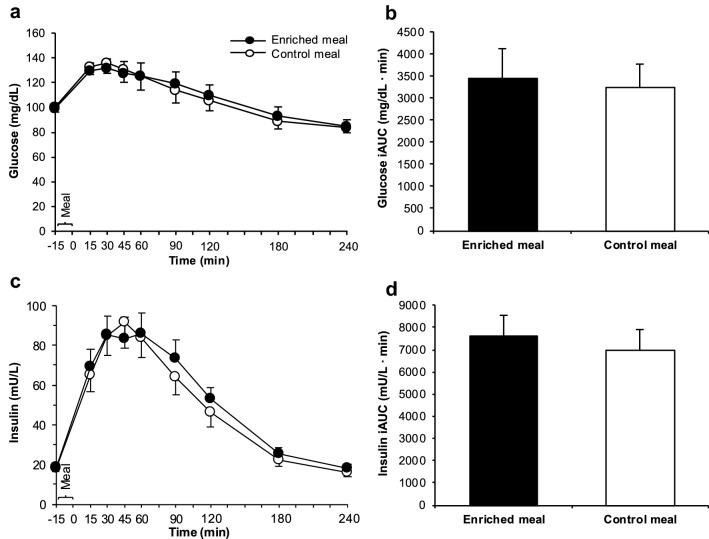
Glucose concentrations (**a**), glucose iAUC (**b**), insulin concentrations (**c**) and insulin iAUC (**d**) after consumption of the enriched meal compared to the control meal. Data: means ± SEMs. Glucose concentrations were analyzed with Friedman test (*P* < 0.001; *n* = 18), followed by Wilcoxon test with Bonferroni correction (*P* ≥ 0.05). Insulin concentrations were analyzed by repeated-measures ANOVA (logarithmized values; effects by time *P* < 0.001, effects by treatment and meal × time interactions *P* ≥ 0.05 each; *n* = 18). iAUCs were compared by paired *t* test (*P* ≥ 0.05 each; *n* = 22). iAUC: incremental area under the curve

**Fig. 4:**
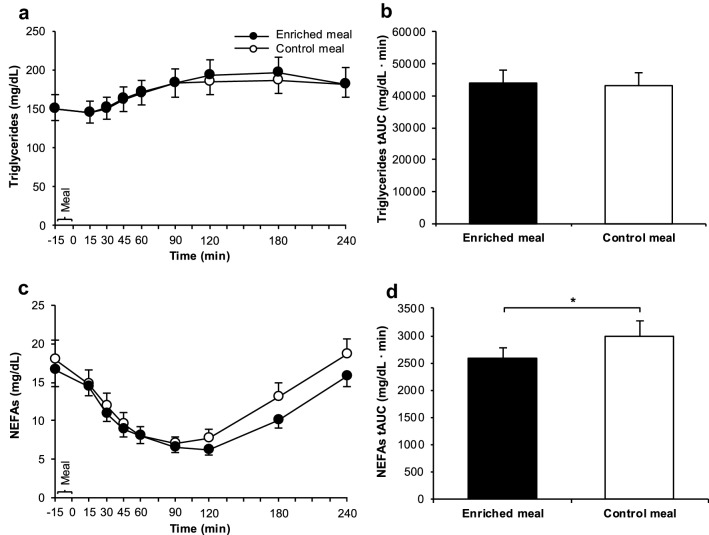
TG concentrations (**a**), TGs tAUC (**b**), NEFA concentrations (**c**) and NEFAs tAUC (**d**) after consumption of the enriched meal compared to the control meal. Data: means ± SEMs. TG concentrations were analyzed with Friedman test (*P* < 0.001; *n* = 18), followed by Wilcoxon test with Bonferroni correction (*P* ≥ 0.05). NEFA concentrations were analyzed with the use of repeated-measures ANOVA (logarithmized values; effects by time *P* < 0.001, effects by treatment and meal × time interactions *P* ≥ 0.05 each; *n* = 18). tAUCs were compared by paired *t* test (*P* ≥ 0.05 for TGs, *P ≤ 0.05 for NEFAs; *n* = 22). NEFAs: non-esterified free fatty acids, tAUC: total area under the curve, TGs: triglycerides

**Fig. 5:**
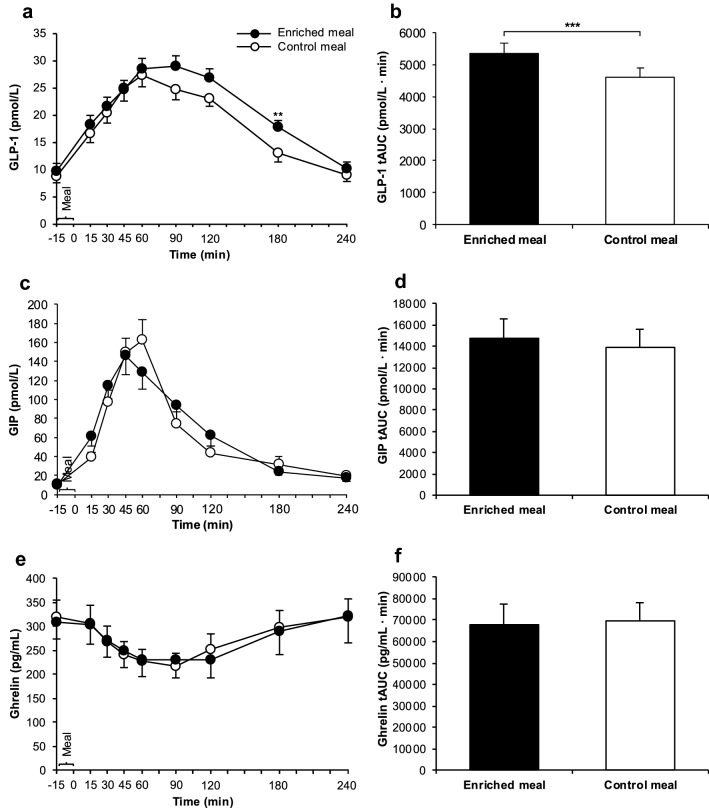
GLP-1 concentrations (**a**), GLP-1 tAUC (**b**), GIP concentrations (**c**), GIP tAUC (**d**), ghrelin concentrations (**e**) and ghrelin tAUC (**f**) after consumption of the enriched meal compared to the control meal. Data: means ± SEMs. During intervention, changes in plasma concentrations of GLP-1 (*n* = 18), GIP (*n* = 18) and ghrelin (*n* = 19) were investigated using Friedman test (*P* < 0.001 for each parameter), followed by Wilcoxon test with Bonferroni correction. *P* values were always ≥ 0.05 if not indicated otherwise. **P ≤ 0.01. tAUCs were compared by paired* t* test (GLP-1: ***P ≤ 0.001; GIP and ghrelin: *P* ≥ 0.05 each; *n* = 22). GIP: gastric inhibitory polypeptide, GLP-1: glucagon-like peptide-1, tAUC: total area under the curve

**Fig. 6:**
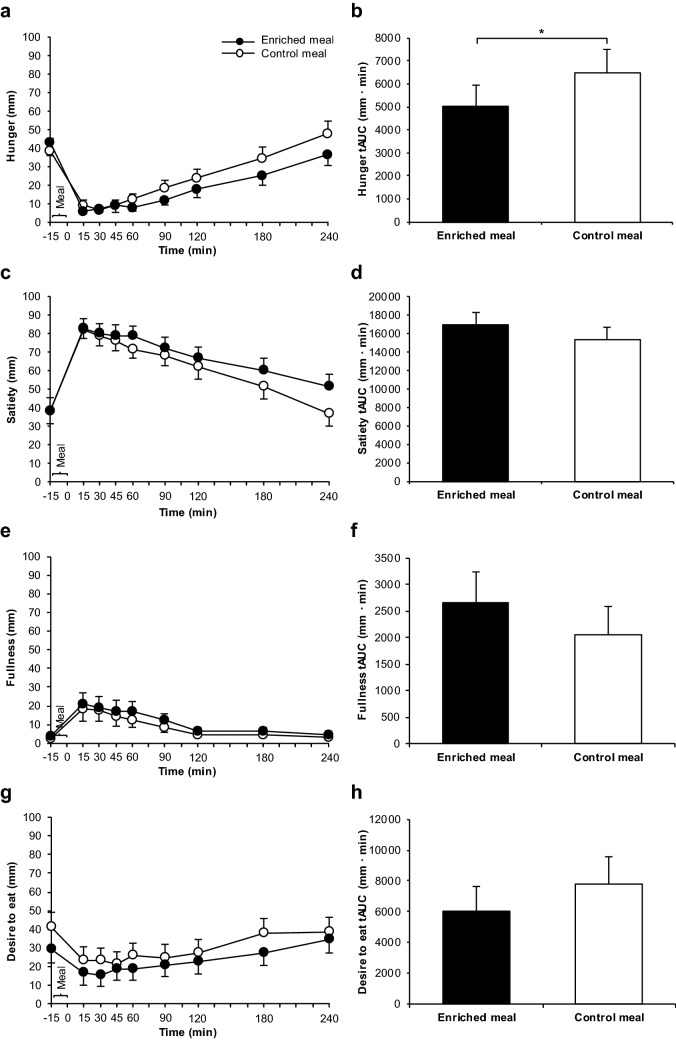
Hunger ratings (**a**), hunger tAUC (**b**), satiety ratings (**c**), satiety tAUC (**d**), fullness ratings (**e**), fullness tAUC (**f**), desire to eat ratings (**g**) and desire to eat tAUC (**h**) after consumption of the enriched meal compared to the control meal. Data: means ± SEMs. All ratings were analyzed using Friedman test (*P* < 0.001 each; *n* = 22), followed by Wilcoxon test with Bonferroni correction (*P* ≥ 0.05 for all comparisons). tAUCs were compared by paired *t* test (hunger: *P ≤ 0.05; further sensations: *P* ≥ 0.05 each; *n* = 22). tAUC: total area under the curve

The baseline characteristics of the participants are shown in Table 2. Nutrition status (BW, BMI, waist circumference, WHR, and FM) was not significantly different before both treatments. Moreover, the mean daily intake of energy and selected nutrients (macronutrients, fatty acid pattern, cholesterol, fiber) was comparable 3 days prior to each intervention (Supplemental Table 2). In fasting state, laboratory parameters (Figs. 3, 4, 5), HOMA-IR (EN: 4.3 ± 0.5 vs. CON: 4.3 ± 0.4, *P* > 0.05 according to *t* test) and appetite sensations (Fig. 6) did not differ before both treatments.

**Table 2:** Baseline characteristics of the participants^a^

	Females (*n* = 14)	Males (*n* = 8)
Age, y	42.7 ± 17.4	47.6 ± 18.2
Height, m	1.68 ± 0.05	1.75 ± 0.08
BW, kg	99.5 ± 18.6	98.0 ± 20.1
BMI, kg/m^2^	35.4 ± 6.3	31.8 ± 5.9
BMI classification^b^		
Normal weight, *n* (%)	1 (7.1)	0 (0.0)
Overweight, *n* (%)	0 (0.0)	3 (37.5)
Obesity, class I, *n* (%)	8 (57.1)	3 (37.5)
Obesity, class II, *n* (%)	2 (14.3)	1 (12.5)
Obesity, class III, *n* (%)	3 (21.4)	1 (12.5)
Fat mass, % BW	46.0 ± 5.2	33.1 ± 5.5
Fat distribution		
Waist circumference, cm	106.4 ± 13.3	109.2 ± 12.7
Waist-to-hip ratio	0.85 ± 0.07	0.99 ± 0.06
Android fat distribution, *n* (%)^b^	7 (50.0)	8 (100.0)
Gynoid fat distribution, *n* (%)^b^	7 (50.0)	0 (0.0)

BW, body weight

^a^Data are means ± SDs unless indicated otherwise. Determined in fasting state at the first visit

^b^According to WHO

### Glucose and insulin

Plasma glucose and serum insulin concentration changed postprandially (*P* ≤ 0.001 each) without significant differences between both treatments at any time (Fig. 3a, c). Postprandial iAUCs for glucose and insulin were not different between EN and CON (Fig. 3b, d). Hence, no treatment effect was detectable (Supplemental Table 3).

### Triglycerides and non-esterified free fatty acids

Postprandially, serum TGs increased and NEFAs decreased by time (*P* < 0.001 each) without any differences between EN and CON (Fig. 4a, c). Moreover, tAUC for TGs was similar for the two meals (Fig. 4b), whereas for NEFAs, tAUC was on average 14% lower after EN compared to CON (*P* = 0.026) (Fig. 4d). Consequently, a treatment effect was found for NEFAs tAUC, but not for TGs tAUC (Supplemental Table 3).

### Gastrointestinal hormones

GLP-1 concentration in plasma increased postprandially (*P* ≤ 0.001) and reached higher values 180 min after EN vs. CON (*P* = 0.009 after Bonferroni adjustment) (Fig. 5a). Moreover, tAUC for GLP-1 was also 17% higher after EN compared to CON (*P* = 0.001; Fig. 5b) and a treatment effect was found (Supplemental Table 3). During intervention, changes in the plasma level of GIP and ghrelin were observed (*P* < 0.001 each) without any significant differences between EN and CON (Fig. 5c, e). No differences in tAUCs for GIP and ghrelin were detectable (Fig. 5d, f) and no treatment effects were found (Supplemental Table 3).

### Appetite sensations

While hunger, satiety, fullness and desire to eat changed after both meals (*P* < 0.001 for all sensations except of fullness with *P* = 0.005), no significant differences between EN and CON were detectable for any of these sensations (Fig. 6a, c, e, g). After EN, tAUC for hunger was about 22% lower than after CON (*P* = 0.031; Fig. 6b) indicating an effect by treatment (Supplemental Table 3). With regard to satiety, the significance for a higher tAUC after EN compared to CON was borderline (*P* = 0.07; Fig. 6d) and no treatment effect was detectable (Supplemental Table 3). Concerning fullness and desire to eat, tAUCs did not differ between both treatments (Fig. 6f, h) and did not indicate any treatment effect (Supplemental Table 3).

### Adverse effects

Few participants reported adverse effects at individual times points (nausea: 1 × EN; headache: 1 × EN, 1 × CON; flatulence: 4 × EN, 2 × CON; heartburn: 2 × EN) without any differences between both treatments (for each adverse effect *P* ≥ 0.05).

## Discussion

To the best of our knowledge, this was the first RCT investigating the overall metabolic and hormonal effects of a usual meal fortified with powder from whole fruiting bodies of dried *P. ostreatus* in adults with IGT. Contrary to our expectations, postprandial glycemic response was not modulated by EN compared to CON. However, EN exerted positive effects on NEFAs in serum, GLP-1 in plasma and on hunger sensation, thereby partly confirming our working hypothesis. EN was well tolerated and may, thus, be qualified to be part of daily diets.

The lack of effects on PBG (Fig. 3a, b) was surprising, but this is consistent with the unchanged insulinemic response (Fig. 3c, d). With 8.1 g of *β*-glucans per 47.6 g avCHO (Table 1), the ratio of *β*-glucan to avCHO was 23% higher than considered for a meal to effectively reduce postprandial glycemic response according to the EFSA Health Claim [8]. This health claim is based on intervention studies with cereal *β*-glucans with a slightly different chemical structure compared to mushroom *β*-glucans [7]. Although earlier in vitro studies with both isolated cereal and mushroom *β*-glucans demonstrate a comparable, structure-independent retardation of starch digestion [23], it cannot be ruled out that these structural differences may explain the lack of PBG effects. The effects of cereal *β*-glucans on PBG are ascribed to the high viscosity of *β*-glucan-containing food slowing down CHO digestion [9] and GE [10]. In a recently published in vitro and in vivo trial [40], an instant oatmeal (1.2 g *β*-glucan) enriched with 10.1 g oat bran (OatWell^®^; 2.8 g *β*-glucans, MW: 2060 kDa) exhibited a 140-fold higher viscosity in vitro than a *β*-glucan-free instant cream of rice used as control (*P* < 0.005); in vivo, the consumption of the enriched meal in healthy adults was associated with a delayed GE and, consequently, a reduction in glucose iAUC compared to the non-enriched cream of rice. Lowering the average MW of *β*-glucans from 2060 kDa to < 10 kDa led only to a fourfold higher viscosity of the instant oatmeal compared to *β*-glucan-free control. As a consequence, no effects on GE and glucose iAUC were observed. The relatively small increase in viscosity of our fortified meal components (soup 0.5-fold and smoothie 1.4-fold higher than non-enriched components) may, thus, explain the lack of changes in PBG. If the increase in the chyme’s viscosity induced by *P. ostreatus* was insufficient to slow down GE, inhibit digestion and thereby decrease the absorption of glucose and fatty acids as expected, it might also explain the lack of changes in TGs (Fig. 4a, b), GIP (Fig. 5c, d), and ghrelin (Fig. 5e, f).

It is conceivable that the mushroom *β*-glucans’ ability to form viscous solutions might be limited if these are provided from oven-dried fruiting bodies. First, in mushroom fruiting bodies, *β*-glucans are in part covalently bound to chitin in the fungal cell wall [41]. Consequently, the luminal solubility of *β*-glucans provided using untreated mushroom powder might have been too low to increase the chyme’s viscosity. Mizutani et al. [24] fortified a fat-based soup with a mushroom extract (obtained by water extraction of oven-dried *P. eryngii* fruiting bodies*)*; after consumption they found lower TG concentrations 3 h and 4 h postprandially (both − 21%, *P* < 0.05) and a lower TGs tAUC (− 18%, *P* < 0.05) compared to the non-enriched soup. The extraction process probably released water-soluble *β*-glucans, which may have increased the viscosity of chyme. Second, the marginal increase in meal viscosity could be explained by the technology to produce mushroom powder. Our mushroom powder was produced by oven-drying; alternatively, mushroom powder can be obtained by lyophilization. Recently, a lower rehydration capacity of oven-dried *P. ostreatus* fruiting bodies compared to a corresponding lyophilized product was observed which was explained by changes of the microstructural properties (e.g., formation of hardened layers) during the different drying processes [42]. Jayasuriya et al. [26] provided 75 g pure glucose to patients with T2DM after a preload of lyophilized *P. ostreatus* fruiting bodies and observed, in contrast to the present study, a lowered 2 h PBG.

At first glance, it seems contradictory that the NEFAs tAUC was reduced (Fig. 4d) after EN compared to CON despite the lack of changes in TGs and insulin. Postprandial NEFAs particularly originate from hydrolysis of circulating TGs by lipoprotein lipase or from hydrolysis of adipose tissue TGs by hormone-sensitive lipase; both enzyme activities are modulated by insulin [43]. Thus, the reduction in NEFAs tAUC after EN vs. CON may be explained by another mechanism possibly lowering lipolysis in adipose tissue and subsequently the efflux of NEFAs: G protein-coupled receptors 43 (GPR43) found in adipocytes are known to bind short-chain fatty acids (SCFAs; acetate, propionate, butyrate) produced by microbial fermentation of dietary fiber. As reviewed recently, the activation of GPR43 in adipocytes may inhibit lipolysis in adipose tissue in both in vitro and animal studies [44]. In mice, the expression of GPR43 in adipocyte tissue increased after feeding a high-fat diet enriched with 5% polysaccharides from *P. eryngii* for 16 weeks compared to a non-enriched diet [45]. Moreover, GPR43 are also expressed on the basolateral side of enteroendocrine L cells and binding SCFAs may stimulate the synthesis of GLP-1 [44]. Therefore, the decrease in NEFAs (Fig. 4d) and the increase in GLP-1 (Fig. 5a, b) might have been induced by SCFAs originating from microbial fermentation of *β*-glucans. Whether microbial metabolites from fungal *β*-glucans may affect postprandial metabolism in humans is unclear; this may be rather expected to occur by regular intake of fermentable fiber. However, the co-ingestion of inulin with pure glucose [46] or high-fructose corn syrup [47] has shown to increase acetate [46, 47] and butyrate [46] in plasma already within 4 h (tAUC_0-4 h_) compared to inulin-free control. These changes were accompanied by lower serum concentration of NEFAs 4 h postprandially [47]. Our assumption that SCFAs might have modulated the GLP-1 and NEFAs response in plasma/serum is supported by the fact that the drifting of both curves (Figs. 4c, 5a) after EN and CON becomes obvious 3 h postprandially.

An obvious explanation for the decrease in hunger by EN vs. CON with respect to tAUC (Fig. 6b) may be the GLP-1-mediated actions of the paracrine and endocrine system, namely that stimulation of GLP-1 receptors located in submucosal vagal afferent nerves and in the brain can modulate appetite sensations [48]. The lack of differences in fullness (Fig. 6e, f) and desire to eat (Fig. 6g, h) appears plausible as the volume of the enriched and non-enriched meal components was similar and the glycemic response did not differ between the meals.

If AUCs between both treatments were compared for women (*n* = 14) and men (*n* = 8) separately, significant differences were additionally found for ghrelin and satiety in women. Ghrelin tAUC (pg/mL ∙ min) was lower [mean difference (95% CI) − 5461(− 9239; − 1683); *P* = 0.008] and tAUC for satiety (mm ∙ min) was higher [mean difference (95% CI) 3175 (1584; 4766); *P* = 0.001] after EN vs. CON. It remains open if such differences did not occur in men or if they were not detectable due to the relatively low sample size.

The strengths of our human intervention study are its double-blinded design, the strong control of confounding factors, such as lifestyle, nutrition status and hormonal variations across menstrual cycle, the detailed characterization of the nutrient composition of the meal components including the mushroom powder, and the determination of physicochemical properties of the meals. An obvious limitation is the fact that our sample size calculation was based on the results of an intervention study with cereal *β*-glucans (oat bran) [37]. Since mushroom *β*-glucans are partly cross-linked with chitin [41], their water-solubility and, thus, their efficacy might be lower compared to cereal-*β*-glucans. Consequently, the expected decrease in glucose iAUC by fortifying a meal with mushroom powder might have been overestimated which means that an effect on glucose iAUC cannot be ruled out. Our working hypothesis included the idea to qualify dried mushrooms as “meal fortifier” and not to fully replace other meal constituents (e.g., wheat flour). Consequently, the macronutrient composition of the test meals varied slightly: EN provided 5.5 g more avCHO than CON (20 g *P. ostreatus* powder: 4.2 g trehalose, 0.9 g *α*-glucans, 0.4 g other mono-/disaccharides and polyols). However, avCHO such as trehalose, and polyols, like mannitol, exhibit no or low glycemic and/or insulinemic effects [49, 50] and probably did not bias our results.

In conclusion, the short-term postprandial metabolic changes observed in this RCT indicate that an easily manageable fortification of a single meal with powder from dried mushroom as a *β*-glucan source may help to attenuate repeated postprandial metabolic deterioration and, in the long run, reduce the known risk for development of overweight/obesity and T2DM in subjects with IGT. Thus, the fortification of meals with mushroom powder might be an alternative to enrichment with oats or barley. Several factors influencing the efficacy of mushroom powder (e.g., *β*-glucan/avCHO ratio, dosage, preparation) should be the focus of future human intervention studies.

## Supplementary Information

Below is the link to the electronic supplementary material.

Supplementary file1 (DOCX 54 KB)

## Data Availability

Data described in the manuscript will not be made available as the participants were assured in the informed consent form that personal data will not be disclosed to third parties.
