# Supplementary material for: Fortifying a meal with oyster mushroom powder beneficially affects postprandial glucagon-like peptide-1, non-esterified free fatty acids and hunger sensation in adults with impaired glucose tolerance: a double-blind randomized controlled crossover trial
Source: Eur J Nutr. 2021 Sep 10;61(2):687–701. doi: 10.1007/s00394-021-02674-1 (PMC8854321; doi:10.1007/s00394-021-02674-1)
Supplement: Supplementary file 1 — Supplementary file1 (DOCX 54 KB) [file 394_2021_2674_MOESM1_ESM.docx]

**Dicks et al.: Fortifying a meal with oyster mushroom powder beneficially affects postprandial glucagon-like peptide-1, non-esterified free fatty acids and hunger sensation in adults with impaired glucose tolerance: a double-blind randomized controlled crossover trial**

**Supplemental Table 1:** Pattern of amino acids, fatty acids and carbohydrates in the meals, based on the analyses of their components^1^

|  | Meal components | | | Meal | |
| --- | --- | --- | --- | --- | --- |
|  | Potato soup  (390 mL) | Smoothie  (250 mL) | Mushroom powder (20 g) | Enriched^2^ | Control^3^ |
| Amino acids^4^ |  |  |  |  |  |
| Alanine, mg | 261 | 59 | 149 | 469 | 320 |
| Arginine, mg | 246 | 86 | 122 | 454 | 332 |
| Aspartic acid/Asparagine, mg^5^ | 587 | 299 | 221 | 1107 | 886 |
| Cysteine, mg | 52 | 8 | 24 | 84 | 60 |
| Glutamic acid/Glutamine, mg^6^ | 884 | 117 | 328 | 1329 | 1001 |
| Glycine, mg | 230 | 33 | 113 | 376 | 263 |
| Histidine, mg | 160 | 27 | 424 | 611 | 187 |
| Isoleucine, mg | 205 | 27 | 93 | 325 | 232 |
| Leucine, mg | 353 | 47 | 153 | 553 | 400 |
| Lysine, mg | 333 | 47 | 131 | 511 | 380 |
| Methionine, mg | 86 | 14 | 39 | 139 | 100 |
| Phenylalanine, mg | 213 | 30 | 98 | 341 | 243 |
| Proline, mg | 318 | 90 | 92 | 500 | 408 |
| Serine, mg | 217 | 50 | 121 | 388 | 267 |
| Threonine, mg | 205 | 30 | 114 | 349 | 235 |
| Tryptophan, mg^7^ | 43 | 6 | 28 | 77 | 49 |
| Tyrosine, mg | 137 | 14 | 63 | 214 | 151 |
| Valine, mg | 254 | 38 | 119 | 411 | 292 |
| Fatty acids^8^ |  |  |  |  |  |
| Caproic acid (6:0), % | 0.5 | 0.0 | 0.0 | 0.5 | 0.5 |
| Caprylic acid (8:0), % | 0.4 | 0.0 | 0.0 | 0.4 | 0.4 |
| Capric acid (10:0), % | 0.8 | 0.0 | 0.0 | 0.8 | 0.8 |
| Lauric acid (12:0), % | 1.2 | 2.0 | 0.0 | 1.2 | 1.2 |
| Myristic acid (14:0), % | 4.1 | 7.0 | 0.5 | 4.1 | 4.1 |
| Palmitic acid (16:0), % | 38.0 | 24.0 | 18.0 | 37.6 | 37.9 |
| Stearic acid (18:0), % | 9.0 | 3.0 | 2.0 | 8.8 | 8.9 |
| Oleic acid (18:1ω-9), % | 36.0 | 23.0 | 10.0 | 35.5 | 35.9 |
| Linoleic acid (18:2ω-6), % | 9.0 | 19.0 | 70.0 | 10.0 | 9.1 |
| Linolenic acid (18:3ω-3), % | 1.8 | 21.0 | 0.0 | 1.9 | 2.0 |
| Behenic acid (22:0), % | 0.3 | 1.0 | 0.0 | 0.3 | 0.3 |
| Special carbohydrates |  |  |  |  |  |
| Arabinose, g^9^ | NA | ND | 0.01 | 0.01 | ND |
| Fructose, g^9^ | ND | 10.1 | ND | 10.1 | 10.1 |
| Galactose, g^9^ | ND | ND | ND | ND | ND |
| Glucose, g^9^ | 0.4 | 5.1 | 0.1 | 5.5 | 5.5 |
| Mannose, g^9^ | ND | ND | ND | ND | ND |
| Rhamnose, g^9^ | ND | ND | ND | ND | ND |
| Xylose, g^9^ | ND | ND | ND | ND | ND |
| Lactose, g^9^ | 2.0 | ND | ND | 2.0 | 2.0 |
| Saccharose, g^9^ | 1.4 | 5.7 | ND | 7.1 | 7.1 |
| Ergosterol, mg^10^ | NA | NA | 41.3 | 41.3 | NA |

^1^ Results present mean values, calculated from analyses in duplicate. NA, not analyzed; ND, not detectable

^2^ Calculated as sum of potato soup, smoothie and mushroom powder except for ergosterol.

^3^ Calculated as sum of potato soup and smoothie.

^4^ Determined by HPLC according to Commission Directive 98/64/EC except of tryptophan.

^5^ Detected as aspartic acid as differentiation between asparagine and aspartic acid was not possible.

^6^ Detected as glutamic acid as differentiation between glutamine and glutamic acid was not possible.

^7^ Determined by HPLC after alkaline hydrolysis of proteins.

^8^ Determined by gas chromatography with flame ionization detection.

^9^ Determined by high performance anion exchange chromatography with pulsed amperometric detection.

^10^ Determined by HPLC-DAD as described previously in Ahlborn J, Calzolari N, Spielmeyer A et al (2018) Enrichment of vitamin D2 in mycelium from submerged cultures of the agaric mushroom *Pleurotus sapidus*. J Food Sci Technol 2018; 55:3833–3839. doi: 10.1007/s13197-018-3290-z

**Supplemental Table 2: Nutrition status in fasting state and nutritional daily intake before each treatment^1^**

|  | Before enriched meal  (*n* = 22) | Before control meal  (*n* = 22) | *P* value |
| --- | --- | --- | --- |
| Nutrition status |  |  |  |
| Weight, kg | 98.9 ± 4.0 | 98.9 ± 4.0 | 0.780^2^ |
| BMI, kg/m² | 34.1 ± 1.3 | 34.1 ± 1.3 | 0.747^2^ |
| Waist circumference, cm | 107.5 ± 2.8 | 107.5 ± 2.8 | 0.876^2^ |
| Waist-to-hip ratio | 0.90 ± 0.02 | 0.90 ± 0.02 | 0.425^2^ |
| Fat mass, % BW | 41.2 ± 1.7 | 41.1 ± 1.7 | 0.833^3^ |
| Nutritional intake/d^4^ |  |  |  |
| Energy, kcal | 2032 ± 120 | 2083 ± 177 | 0.748^2^ |
| Protein, g | 78 ± 5 | 83 ± 6 | 0.426^2^ |
| Protein, g/kg BW | 0.8 ± 0.1 | 0.9 ± 0.1 | 0.321^2^ |
| Fat, g | 94 ± 8 | 94 ± 10 | 0.961^2^ |
| SFAs, g | 36 ± 3 | 39 ± 4 | 0.392^2^ |
| MUFAs, g | 32 ± 3 | 34 ± 4 | 0.623^2^ |
| PUFAs, g^5^ | 18 ± 2 | 15 ± 2 | 0.300^2^ |
| Cholesterol, mg | 381 ± 47 | 372 ± 36 | 0.851^2^ |
| Carbohydrates, g^5^ | 205 ± 13 | 212 ± 20 | 0.865^2^ |
| Dietary fiber, g | 19 ± 1 | 18 ± 1 | 0.544^2^ |
| Water soluble, g | 6 ± 0 | 6 ± 0 | 0.162^2^ |
| Water insoluble, g | 12 ± 1 | 12 ± 1 | 0.784^2^ |
| Alcohol, g | 0 ± 0 | 0 ± 0 | 0.953^3^ |

^1^ Data are means ± SEMs.

^2^ Determined by *t* test for paired samples.

^3^ Determined by Wilcoxon test.

^4^ Mean daily intake as determined by 3-d dietary records before each treatment.

^5^ Logarithmized values were used for paired *t* test.

BW, body weight

**Supplemental Table 3:** Differences between postprandial AUCs obtained after enriched and control meal^1^

|  | Mean difference (95% CI) | *P* value |
| --- | --- | --- |
| Glucose metabolism |  |  |
| Glucose iAUC, mg/dL ⋅ min^2^ | 234 (-225, 704) | 0.351 |
| Insulin iAUC, mU/L ⋅ min^2^ | 624 (-166, 1583) | 0.209 |
| Lipids |  |  |
| Triglycerides tAUC, mg/dL ⋅ min | 726 (-2720, 4173) | 0.666 |
| NEFAs tAUC, mg/dL ⋅ min | -15 (-28, -2) | 0.026 |
| Gastrointestinal hormones |  |  |
| GLP-1 tAUC, pmol/L ⋅ min | 766 (338, 1194) | 0.001 |
| GIP tAUC, pmol/L ⋅ min^2^ | 839 (-1449, 2913) | 0.472 |
| Ghrelin tAUC, pg/mL ⋅ min^2^ | -1812 (-7136, 5956) | 0.600 |
| Appetite sensations |  |  |
| Hunger tAUC, mm ⋅ min^2^ | -1455 (-2678, -282) | 0.031 |
| Satiety tAUC, mm ⋅ min | 1533 (-107, 3174) | 0.065 |
| Fullness tAUC, mm ⋅ min^2^ | 611 (-26, 1341) | 0.106 |
| Desire to eat tAUC, mm ⋅ min^2^ | -1713 (-4265, 279) | 0.171 |

^1^ Data are based on *n* = 22. Postprandial AUCs were compared by paired *t* test.

^2^ 95% CI was estimated by using bootstrapping.

GIP: gastric inhibitory polypeptide, GLP-1: glucagon-like peptide-1, iAUC: incremental area under the curve, NEFAs: non-esterified free fatty acids, tAUC: total area under the curve
